# Predictive Value of Serum Gamma-glutamyltranspeptidase for Future Cardiometabolic Dysregulation in Adolescents- a 10-year longitudinal study

**DOI:** 10.1038/s41598-017-09719-8

**Published:** 2017-08-29

**Authors:** Chien-Ming Lin, Chang-Hsun Hsieh, Chien-Hsing Lee, Dee Pei, Jiunn-Diann Lin, Chung-Ze Wu, Yao-Jen Liang, Yi-Jen Hung, Yen-Lin Chen

**Affiliations:** 1 Department of Pediatrics, Tri-Service General Hospital, National Defense Medical Center, Taipei, Taiwan; 2 Graduate Institute of Medical Sciences, National Defense Medical Center, Taipei, Taiwan; 3 Division of Endocrinology and Metabolism, Department of Internal Medicine, Tri-Service General Hospital, National Defense Medical Center, Taipei, Taiwan; 4 Department of Internal Medicine, Cardinal Tien Hospital, School of Medicine, Fu-Jen Catholic University, New Taipei City, Taiwan; 5 Division of Endocrinology, Department of Internal Medicine, Shuang-Ho Hospital, Taipei Medical University, New Taipei City, Taiwan Republic of China; 6 Division of Endocrinology and Metabolism, Department of Internal Medicine, School of Medicine, College of Medicine, Taipei Medical University, Taipei, Taiwan Republic of China; 7 Department of Life Science, Graduate Institute of Applied Science and Engineering, College of Science and Engineering, Fu-Jen Catholic University, New Taipei City, Taiwan; 8 Department of Pathology, Cardinal Tien Hospital, School of Medicine, Fu-Jen Catholic University, New Taipei City, Taiwan

## Abstract

Serum gamma-glutamyltransferase (γ-GT) is implicated in the pathogenesis of atherosclerosis and metabolic syndrome (MetS) in adults. The relationships between γ-GT and cardiometabolic dysregulation remains unclear in adolescents. We enrolled 7,072 Taiwanese adolescents and followed them for a median of 6.8 years. The optimal cut-off values (CoVs) of baseline γ-GT to predict future MetS, hypertension (HTN), and type 2 diabetes (T2DM) were determined by receiving operating characteristic (ROC) curve. Using these CoVs, the participants were divided into normal- and high-level groups. Cox proportional hazard analysis was used to calculate hazard ratios (HRs) for the subjects with a high level of γ-GT for the risk of future cardiometabolic dysregulation. Serum γ-GT was significantly higher in the subjects with MetS than in those without MetS at baseline (p < 0.001). The optimal CoVs of γ-GT were 12 U/L for boys and 11 U/L for girls. In multivariate Cox regression analysis, a higher serum γ-GT level increased the risk of future MetS (HRs 1.98 and 2.85 for boys and girls, respectively, both p < 0.001), but not new onset HTN and T2DM. In conclusion, serum γ-GT levels not only demonstrated an excellent correlation with the presence of MetS and also in predicting future MetS in adolescents.

## Introduction

Adolescents have become increasingly obese worldwide during the last three decades^1, 2^. Importantly, obese adolescents are likely to stay obese into adulthood and are more likely to develop non-communicable diseases such as metabolic syndrome (MetS), type 2 diabetes (T2DM) and cardiovascular disease (CVD)^3–7^. Since these diseases are included in the top ten leading causes of death in Taiwan^8^, the early recognition of adolescents at high risk of future cardiometabolic dysregulation and prevention of associated morbidity and mortality are critical public health issues^9, 10^.

The pathogenesis of cardiometabolic dysregulation with regards to genetic and social-environmental factors is unclear, however it probably involves an imbalance between pro- and anti-inflammatory adipocytokines^11^. Increased levels of pro-inflammatory cytokines such as leptin, tumor necrosis factor-α, interleukin-6 (IL-6), IL-1β and decreased levels of anti-inflammatory cytokines such as adiponectin have been demonstrated both in children and adults with MetS^11, 12^. Even though high molecular weight adiponectin and a high leptin-to-adiponectin ratio have been reported to be useful biomarkers in establishing MetS^13^, the limited testing ability in primary care institutes limits their clinical application. With an increasing prevalence of MetS in adolescents^14^, identifying easy and reliable biomarkers to predict cardiometabolic dysregulation and understanding the relationships between these biomarkers and cardiometabolic dysregulation are also important.

Gamma-glutamyltranspeptidase (γ-GT) is a liver enzyme that participates in the synthesis and degradation of glutathione as well as xenobiotic detoxification^15, 16^. Serum γ-GT is a widely used biomarker for alcoholic liver injury and nonalcoholic fatty liver disease (NAFLD). Previous studies have also reported the diagnostic role of serum γ-GT in MetS, T2DM, and CVD, and its predictive role of mortality and morbidity associated with cardiometabolic dysregulation^17–20^. However, these studies only enrolled middle-aged patients, and thus cannot be extrapolated to adolescents^17–20^. A recent cohort study recruiting 1,874 adolescents demonstrated that the subjects with NAFLD had higher γ-GT levels and greater liver shear velocity (an indicator of liver fibrosis) than those without NAFLD, even after adjustment for fat mass^21^. Although the association between serum γ-GT and ultrasound scan-determined liver damage was identified^21^, the cross-sectional study cannot determine the causality. In addition, the role of γ-GT in future cardiometabolic dysregulation is also uncertain in adolescents. This longitudinal study aimed to evaluate the relationships between baseline γ-GT levels and MetS and its component, and to assess whether optimal cut-off values (CoVs) of γ-GT can predict future MetS, hypertension (HTN) and T2DM in adolescents.

## Methods

This study was approved by the Ethical Committee of the Cardinal Tien Hospital and the Ethical Committee of MJ Health Screening Centers. Each participant provided written informed consent. The described methods were carried out in accordance with the guidelines of the Declaration of Helsinki.

### Study Participants

**Figure 1:**
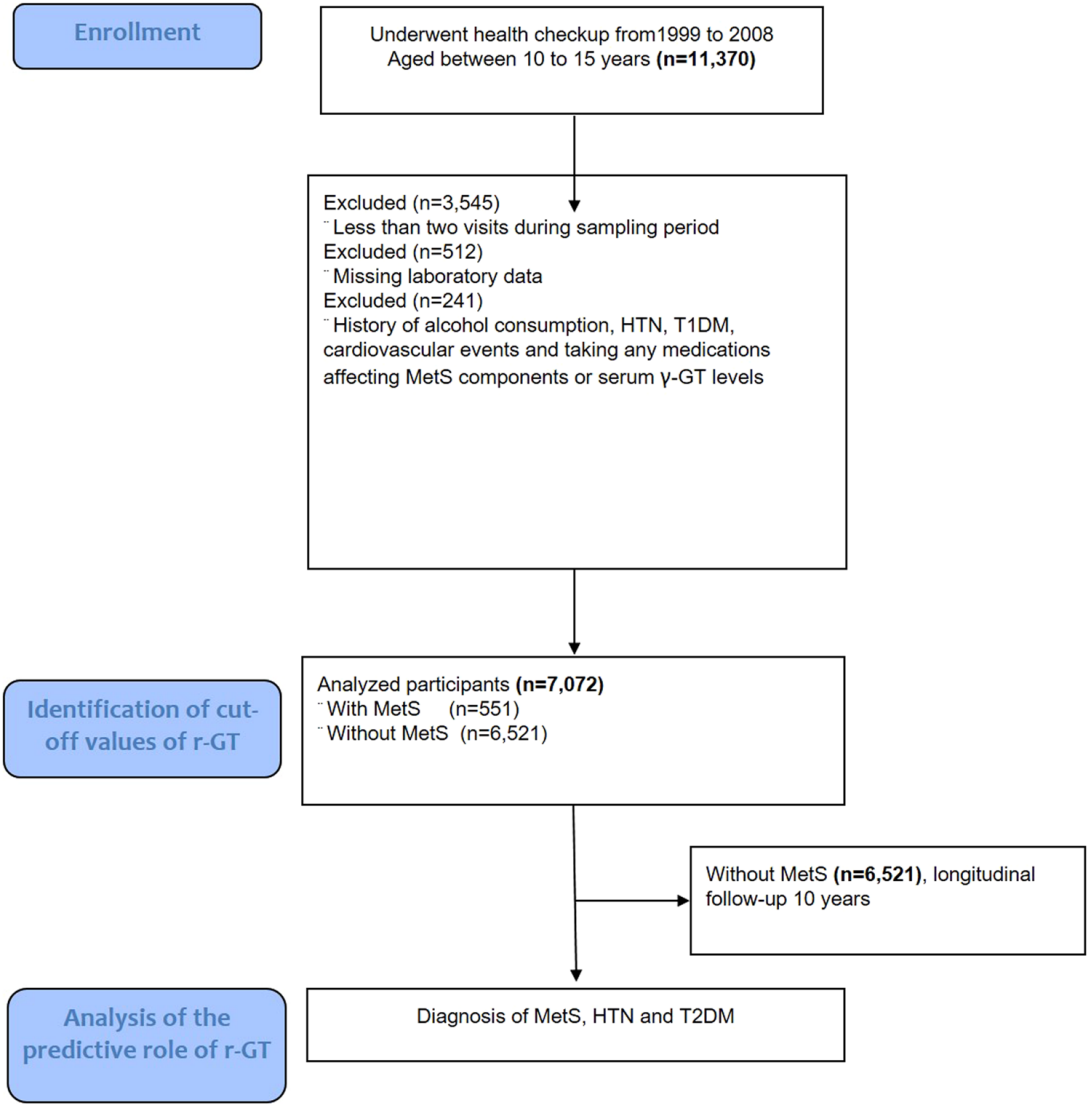
Enrollment flow diagram. A total of 11,370 participants aged from 10 to 15 years who underwent regular health examinations from 1999 to 2008 at MJ Health Screening Centers were enrolled. Among them, the subjects with only one visit (n = 3,545), missing data of MetS components or γ-GT (n = 512), and a history of alcohol consumption, HTN, type 1 diabetes and those taking medications known to affect MetS components or serum γ-GT levels (n = 241) were excluded. The remaining 7,072 subjects were enrolled as the study cohort. In stage 1, the optimal CoVs of baseline γ-GT to differentiate the subjects with and without MetS were identified by ROC curve. Using these CoVs, the aim of second stage was to validate its predictive role on future MetS, HTN and T2DM.

We enrolled subjects from MJ Health Screening Centers, a privately-owned chain of clinics throughout Taiwan which provide regular health examinations to their members. Parental informed consent and assent form the young adolescents were obtained. Data from the participants were collected anonymously and provided for research purposes only. In total, 11,370 subjects aged from 10 to 15 years were enrolled during a 10-year sample period (1999 to 2008) (Fig. 1). The exclusion criteria were those with only one visit (n = 3,545), those with missing data of MetS components or γ-GT (n = 512) and those with a history of alcohol consumption, HTN, type 1 diabetes and those taking medications known to affect MetS components or serum γ-GT levels including antihypertensive agents, corticosteroid, glycemic control agent, antilipid agent, antipsychotics, antidepressants, antiepileptics and immunosuppressants (n = 241). The remaining 7,072 subjects (3,954 boys and 3,118 girls) were enrolled as the study cohort.

### Study Design

There are two parts to this study. The first was a cross-sectional observation on the relationships between baseline γ-GT levels and MetS and its components. In addition, the optimal CoVs of baseline γ-GT to differentiate the subjects with and without MetS were identified. The second stage of this study was longitudinal. The primary aim of this stage was to validate the CoVs determined in stage 1. Thus, 551 subjects who had MetS at baseline were excluded, and the remaining 6,521 subjects without MetS were followed up annually with the range of 2 to 10 years (median 6.8 years). Based on the γ-GT CoVs, we grouped the subjects without MetS into those with normal- and high-levels of γ-GT. The incidence rates of developing future MetS, HTN and T2DM were then calculated in the two groups.

### General Data and Anthropometric Measurements

The senior nursing staff used a questionnaire to obtain the subjects’ drinking habits and medical history. Complete physical examinations were then performed. Anthropometric measurement including waist circumference (WC), body weight, body height, systolic blood pressure, and diastolic blood pressure were measured as we described previously^22–24^. After 10-hour fasting, blood samples were drawn from the antecubital vein for biochemical analysis. Plasma was separated from the blood within 1 hour and stored at −30 °C until fasting plasma glucose (FPG) and lipid profile analysis. The FPG was detected using a glucose oxidase method (YSI 203 glucose analyzer, Scientific Division, Yellow Springs Instruments, Yellow Springs, OH). Total cholesterol, triglycerides (TG), and low-density lipoprotein cholesterol (LDL-C) concentrations were measured by an enzymatic colorimetric method with a Roche Cobas C501 Chemistry Analyzer (Diamond Diagnostics, USA). Serum levels of high-density lipoprotein cholesterol (HDL-C) were determined using an enzymatic colorimetric assay after dextran sulfate precipitation. Serum γ-GT levels were measured using a CX7 biochemistry analyzer (Beckman, Fullerton, CA)^22–24^.

### Definition of Metabolic Syndrome

We used the International Diabetes Federation (IDF) consensus definition of MetS in children and adolescents to define MetS^22, 25^. Subjects having three or more of the following abnormalities were diagnosed with MetS: abdominal obesity (WC ≥ 90^th^ percentile)^26^, TG ≥ 150 mg/dL, HDL-C < 40 mg/dL, HTN (systolic blood pressure ≥130 or diastolic blood pressure ≥85 mmHg), and FPG concentration ≥100 mg/dL^22^.

### Statistical Analysis

Anthropometric and biochemical data were expressed as mean ± standard deviation. All data were tested for normal distribution using the Kolmogorov-Smirnov test and homogeneity of variance with Levene’s test. The *t*-test was used to evaluate differences in demographic data between the subjects with and without MetS. Univariate and multivariate regression analyses were used to assess correlations between γ-GT and MetS components. The optimal CoVs of γ-GT for a higher likelihood of developing cardiometabolic dysregulation was calculated using receiver operating characteristic (ROC) curve analysis (MedCalc Software, Broekstraat, Mariakerke, Belgium).

In stage 2, hazard ratios (HRs) of having MetS, HTN and T2DM were calculated using Cox regression analysis. In addition, Kaplan-Meier plots and the log rank test were performed to evaluate the time effect on the incidence of having MetS, HTN and T2DM between the two groups. All data were analyzed using SPSS 18.0 software (SPSS Inc., Chicago, IL). A *p*-value (two-sided) < 0.05 was considered to be statistically significant.

## Results

### Baseline Characteristics and Association between γ-GT and MetS

The baseline demographic data of the participants with and without MetS are shown in Table 1. Of the 3,954 male subjects, 332 (8.4%) with a mean age of 13.31 ± 1.97 years and 219 (7.0%) of 3,118 females with a mean age of 13.47 ± 1.96 years fulfilled the diagnostic criteria of MetS. There were significant differences in all five components of MetS (WC, blood pressure, FPG, HDL-C, and TG) between the subjects with and without MetS in both genders. Notably, the level of serum γ-GT was significantly higher in the subjects with MetS than in those without (*p* < 0.001).

**Table 1:** Demographic data of the study subjects with and without metabolic syndrome at baseline.

	Male					Female				
	MetS (−)		MetS (+)		P value	MetS (−)		MetS (+)	P value	
n	3622		332			2899		219		
Age (years)	13.2	±2.0	14.0	±1.7	<0.001	13.5	±2.0	13.2	±1.9	0.031
Waist circumference (cm)	68.6	±10.1	82.9	±10.7	<0.001	63.8	±7.4	72.8	±9.2	<0.001
Systolic blood pressure (mmHg)	110.7	±12.7	127.3	±13.1	<0.001	105.1	±11.5	115.3	±14.8	<0.001
Diastolic blood pressure (mmHg)	60.5	±8.6	67.5	±10.1	<0.001	59.1	±7.7	62.2	±8.7	<0.001
Fasting plasma glucose (mg/dl)	94.7	±8.2	99.9	±8.0	<0.001	91.9	±9.4	98.6	±16.4	<0.001
Total cholesterol (mg/dl)	163.5	±29.0	167.6	±34.4	<0.036	166.8	±27.6	170.9	±33.3	0.079
High density lipoprotein (mg/dl)	56.2	±12.9	42.6	±10.3	<0.001	58.1	±12.8	44.1	±8.8	<0.001
Low density lipoprotein (mg/dl)	92.0	±25.4	98.2	±27.7	<0.001	93.6	±24.5	97.8	±29.1	0.038
Triglyceride (mg/dl)	76.3	±35.2	133.6	±65.9	<0.001	75.7	±30.2	145.4	±70.3	<0.001
γ-GT (U/L)	14.1	±7.7	20.3	±13.5	<0.001	10.5	±4.7	13.8	±8.7	<0.001

Data are shown as mean ± SD. Abbreviations: MetS, metabolic syndrome; MetS(−), without metabolic syndrome; MetS(+), with metabolic syndrome; γ-GT, gamma-glutamyl transferase.

Univariate regression analysis showed a significant correlation between γ-GT and all five components of MetS in the males, however, only WC, blood pressure and TG were associated with γ-GT in the females (Table 2). In multivariate regression analysis, WC, HDL-C and TG in the males and WC and TG in the females remained significantly associated with γ-GT levels.

**Table 2:** Univariate and multivariate regression analysis of the γ-GT and components of the metabolic syndrome

	Univariate		Multivariate			
	γ	p	Model 1		Model 2	
			β	p	β	p
Male						
Waist circumference	0.455	<0.001	0.418	<0.001	0.397	<0.001
Systolic blood pressure	0.221	<0.001	0.026	0.141	0.019	0.285
Diastolic blood pressure	0.140	<0.001	0.014	0.380	0.013	0.406
Fasting Plasma Glucose	0.060	<0.001	0.024	0.082	0.026	0.066
High density lipoprotein	−0.112	<0.001	0.110	<0.001	0.118	<0.001
Triglyceride	0.301	<0.001	0.172	<0.001	0.167	<0.001
Female						
Waist circumference	0.220	<0.001	0.170	<0.001	0.163	<0.001
Systolic blood pressure	0.124	<0.001	0.054	0.009	0.051	0.014
Diastolic blood pressure	0.059	0.001	−0.001	0.941	−0.001	0.946
Fasting Plasma Glucose	0.010	0.568	—	—	—	—
High density lipoprotein	−0.012	0.491	—	—	—	—
Triglyceride	0.195	<0.001	0.154	<0.001	0.152	<0.001

Model 1: Adjusted for components of metabolic syndrome. Model 2: Adjusted for components of metabolic syndrome as well as age and low-density lipoprotein.

**Figure 2:**
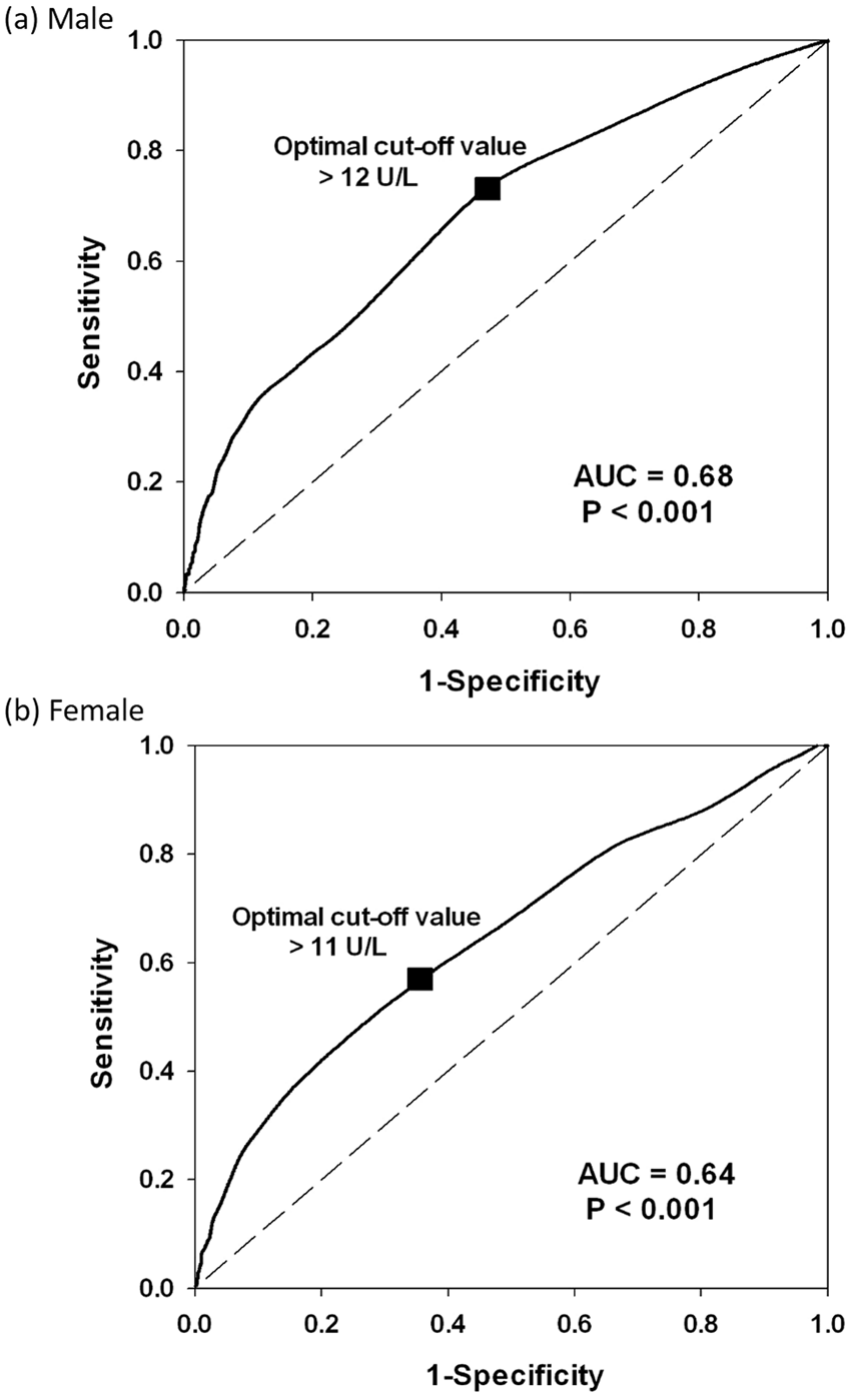
Receiver operating characteristic curves for serum γ-GT in both genders. Receiver operating characteristic curves and optimal cut-off values for serum γ-GT for differentiating between MetS and non-MetS in (**a**) male and (**b**) female adolescents.

ROC curve analysis showed that the optimal CoVs of γ-GT were 12 U/L in males and 11 U/L in females (Fig. 2). The areas under the ROC curve were 0.68 for the males (sensitivity 74.1%, specificity 52.0%) and 0.64 for the females (sensitivity 60.3%, specificity 60.2%) (both *p* < 0.001).

### γ-GT in Predicting Future MetS, HTN, and T2DM

**Figure 3:**
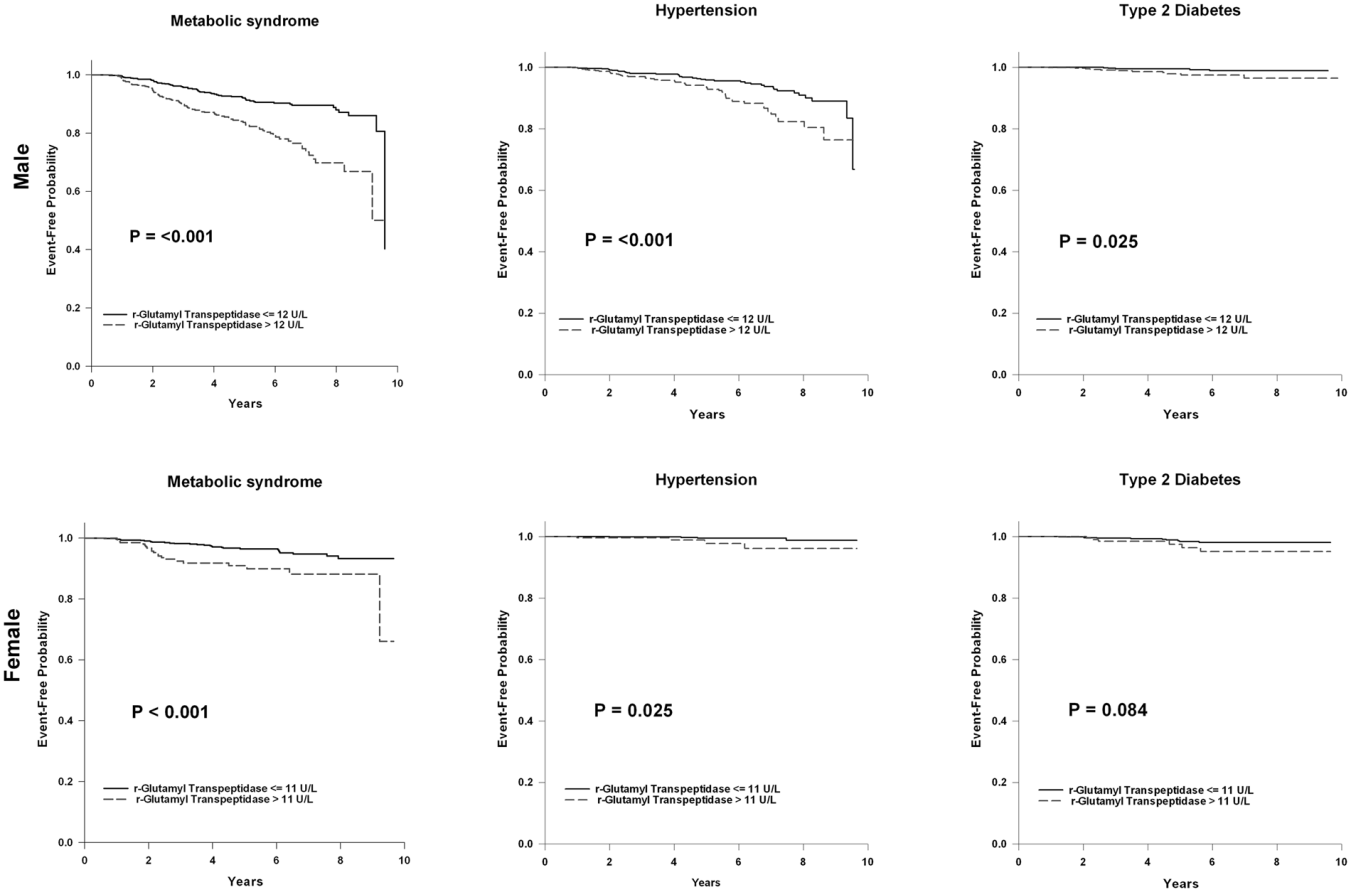
Kaplan-Meier plot of developing future MetS, HTN and T2DM by different γ-GT levels. Kaplan-Meier curves estimate with log rank test was applied for the event-free probability between the subjects with normal γ-GT levels (≤12 U/L) and high γ-GT levels (>12 U/L).

In univariate Cox regression analysis, the subjects with higher baseline levels of γ-GT (>12 U/L in males, >11 U/L in females) had a higher risk of developing MetS and HTN in both genders, and T2DM in males during the follow-up period (median 6.8 years) (Table 3). In addition, multivariate Cox regression analysis showed that a higher serum γ-GT level remained a significant risk factor for future MetS (HR 1.98, 95% confidence interval (CI) 1.42–2.77 in males; HR 2.85, 95% CI 1.60–5.08 in females, both *p* < 0.001), but not in new-onset HTN orT2DM. Kaplan-Meier plots also demonstrated the same findings (Fig. 3).

**Table 3:** Hazard ratios of γ-GT and components of the metabolic syndrome in developing future metabolic syndrome, hypertension and type 2 diabetes.

	Univariate Cox Regression			Multivariate Cox Regression		
	Hazard Ratio (95% CI)		p value	Hazard Ratio (95% CI)		p value
**(a) Metabolic syndrome**						
Male						
γ-GT > 12 U/L	2.526	(1.824–3.500)	<0.001	1.980	(1.417–2.765)	<0.001
WC > criteria*	4.508	(2.919–6.961)	<0.001	3.881	(2.481–6.069)	<0.001
BP > criteria*	1.199	(0.740–1.942)	0.461	0.915	(0.562–1.489)	0.721
FPG > 100 mg/dl	1.517	(1.068–2.156)	0.020	1.437	(1.008–2.048)	0.045
HDL-C<criteria*	1.067	(0.689–1.652)	0.771	0.925	(0.593–1.444)	0.732
TG > 150 mg/dl	1.184	(0.641–2.185)	0.590	0.746	(0.401–1.390)	0.357
Female						
γ-GT > 11 U/L	2.793	(1.589–4.910)	<0.001	2.850	(1.598–5.082)	<0.001
WC > criteria*	1.859	(1.025–3.373)	0.041	1.547	(0.840–2.850)	0.162
BP > criteria*	0.522	(0.072–3.783)	0.520	0.362	(0.049–2.674)	0.320
FPG > 100 mg/dl	1.204	(0.477–3.041)	0.694	1.220	(0.483–3.080)	0.674
HDL-C<criteria*	2.940	(1.669–5.178)	<0.001	2.880	(1.625–5.104)	<0.001
TG > 150 mg/dl	0.930	(0.226–3.831)	0.920	0.422	(0.099–1.793)	0.242
**(b) Hypertension**						
Male						
γ-GT > 12 U/L	2.068	(1.339–3.194)	0.001	1.551	(0.989–2.433)	0.056
WC > criteria*	3.092	(1.816–5.265)	<0.001	2.548	(1.459–4.449)	0.001
BP > criteria*	3.281	(2.032–5.299)	<0.001	2.660	(1.624–4.355)	<0.001
FPG > 100 mg/dl	0.845	(0.484–1.477)	0.554	0.732	(0.416–1.288)	0.279
HDL-C<criteria*	1.273	(0.745–2.175)	0.377	0.941	(0.543–1.632)	0.828
TG > 150 mg/dl	1.544	(0.773–3.084)	0.218	0.913	(0.447–1.864)	0.803
Female						
γ-GT > 11 U/L	4.312	(1.069–17.395)	0.040	2.351	(0.517–10.693)	0.269
WC > criteria*	5.955	(0.732–48.428)	0.095	3.333	(0.379–29.288)	0.278
BP > criteria*	14.494	(3.457–60.769)	<0.001	8.210	(1.830–36.835)	0.006
FPG > 100 mg/dl	1.541	(0.189–12.582)	0.686	1.119	(0.130–9.627)	0.919
HDL-C<criteria*	1.810	(0.452–7.243)	0.402	1.141	(0.253–5.143)	0.864
TG > 150 mg/dl	7.387	(1.477–36.935)	0.015	2.893	(0.448–18.663)	0.264
**(c) Diabetes**						
Male						
γ-GT > 12 U/L	3.165	(1.094–9.160)	0.034	2.429	(0.812–7.266)	0.112
WC > criteria*	5.224	(1.187–22.985)	0.029	4.023	(0.875–18.496)	0.074
BP > criteria*	1.193	(0.271–5.250)	0.815	0.785	(0.175–3.522)	0.752
FPG > 100 mg/dl	1.755	(0.610–5.055)	0.297	1.654	(0.568–4.819)	0.356
HDL-C<criteria*	1.331	(0.379–4.679)	0.656	1.107	(0.303–4.051)	0.878
TG > 150 mg/dl	1.871	(0.425–8.234)	0.407	1.046	(0.224–4.882)	0.955
Female						
γ-GT > 11 U/L	2.378	(0.862–6.561)	0.094	2.757	(0.980–7.755)	0.055
WC > criteria*	0.824	(0.309–2.196)	0.699	0.742	(0.271–2.033)	0.562
BP > criteria*	1.667	(0.220–12.619)	0.621	1.555	(0.199–12.164)	0.674
FPG > 100 mg/dl	0.672	(0.089–5.091)	0.700	0.680	(0.090–5.161)	0.710
HDL-C<criteria*	1.109	(0.403–3.052)	0.841	1.236	(0.444–3.437)	0.685
TG > 150 mg/dl	0.046	(0.000–1178.588)	0.553	0.000	(0.000–—)	0.978

BP, blood pressure; CI, confidence interval; FPG, fasting plasma glucose; γ-GT, gamma-glutamyltranspeptidase; HDL-C, high-density lipoprotein cholesterol; WC, waist circumference. *Criteria for WC were according to the cut-off value by Sung *et al*.^26^; criteria for BP were systolic BP ≥ 130 mmHg or diastolic BP ≥ 85 mmHg; criteria for HDL-C was<40 mg/dL. BP, blood pressure; CI, confidence interval; FPG, fasting plasma glucose; γ-GT, gamma-glutamyltranspeptidase; HDL-C, high-density lipoprotein cholesterol; WC, waist circumference. *Criteria for WC were according to the cut-off value by Sung *et al*.^26^; criteria for BP were systolic BP ≥ 130 mmHg or diastolic BP ≥ 85 mmHg; criteria for HDL-C was <40 mg/dL.

## Discussion

The results of this study revealed that the adolescents with MetS not only had higher γ-GT levels, but also a significant association between γ-GT and MetS, particularly WC and TG. These findings suggest that γ-GT may be involved in the pathophysiology of MetS in adolescents. In accordance with this hypothesis, our longitudinal results over a median follow-up period of 6.8 years indicated that a high serum γ-GT level was an independent predictor for future MetS in adolescents. To the best of our knowledge, this is the first large-scale longitudinal study focusing on adolescents to investigate the role of γ-GT on future MetS, HTN and diabetes in the same time.

Since it is well-known that central obesity and insulin resistance are at the core of MetS, the role of γ-GT in the pathogenesis of MetS might be linked through NAFLD. In subjects with NAFLD, overproduction of glucose and TG from the fatty liver may precipitate the occurrence of MetS. On the other hand, the NAFLD is considered the hepatic manifestation of MetS and commonly associated with obesity^27^. Therefore, NAFLD was reported to be a useful predictor of MetS^28^. Conversely, patients with MetS have an increased risk of developing NAFLD^29^. The highly increasing prevalence of T2DM, obesity, and lifestyle changes (mainly exercise withdrawal) in the general population also makes NAFLD the most common diagnosis in daily clinical practices^30^. Even though NAFLD as a cause or a consequence of MetS is still being debated, an elevated level of γ-GT secondary to excessive liver fat accumulation has been demonstrated in patients with MetS and NAFLD^17^. As expected, γ-GT has been reported to be a surrogate marker of NAFLD, and also a promising biomarker for MetS and its components in adults^19, 20^.

However, little is known about the associations of γ-GT concentration with MetS and the role of γ-GT as features of MetS in adolescents. To elucidate this uncertainty, Kong *et al*. enrolled 2,067 healthy Hong Kong participants aged 6–20 years and demonstrated that high γ-GT levels were associated with components of MetS, especially obesity and high blood pressure^31^. Even though these striking findings support the assumption that serum γ-GT might be a potential predictor for MetS in the youth population, the cross-sectional study could not provide information regarding the temporal and causal relationship between γ-GT and MetS^31^. The present study taking advantage of large-scale longitudinal follow-up aimed to assess the predictive value of γ-GT on future MetS in adolescent males and females. Interestingly, our results showed that γ-GT levels were distinctly associated with the WC, HDL-C and TG components of MetS in the males, but only WC and TG in the females. Similarly, previous studies also suggested differences in age and gender in the way MetS is expressed in adults^32^ as in adolescents^33^. Even though the phenotype of MetS determined by gender was identifiable, we found that a hypertriglyceridemic waist (HTGW) was strongly related to γ-GT levels in both genders, suggesting that HTGW is a useful index for metabolic dysregulation^33–35^. In addition to γ-GT, our results also showed that WC in males and HDL-C in females had predictive power for new-onset MetS. These findings reinforce the hypothesis that MetS is a heterogeneous condition, so that the predictive parameters of MetS in affected subjects can be influenced by age, gender, and race/ethnicity^36^. Taken together, our compelling findings not only identify the relationships between γ-GT, current MetS and future MetS, but also validate differences in gender in the variable expression of MetS in adolescents^31, 33, 35^.

Emerging evidence has revealed the association between NAFLD and increasing odds of MetS^24^. The risk reduction of MetS may be achieved by lowering liver fat. Although pharmacologic therapies for NAFLD remains unavailable^37^, lifestyle interventions such as dieting and exercise have been considered effective^38^. In regard to exercise, Keating *et al*. reported that aerobic exercise training may help to burn fat in liver and viscera regardless of aerobic exercise dose or intensity^39^. Another study on resistance exercise also demonstrated that resistance training lead to a significant reduction in liver fat content and a greater glycemic control in the meanwhile^40^. Even though the existing evidences all supported the role of exercise on improving NAFLD and MetS^38–40^, the precise mechanisms were still unclear. On the other hand, the measurement of serum γ-GT level was a less expensive, widely available and easily interpretable way in primary care institutes to predict MetS, compared to ultrasound scan-determined NAFLD^21^. Considering the cost-effectiveness, the CoVs of γ-GT provided in the present study might be a useful tool to evaluate the long-term efficacy of exercise on NAFLD and MetS, and to clarify their relationships, at least in Taiwanese adolescents.

Although the detailed mechanisms that the link γ-GT with HTN and atherosclerotic CVD remain elusive, there are some possible explanations for their relationships^41, 42^. Previous studies showed that γ-GT is significantly related to markers of inflammation such as fibrinogen, C-reactive protein and F2-isoprostanes^42, 43^. Furthermore, γ-GT is thought to be involved in the pathogenesis of atherosclerosis on the basis of expression of γ-GT in human atherosclerotic lesions^43, 44^. Additionally, the activity of ectoenzymatic γ-GT has been reported to play a pivotal role in the generation of free radical species through modulating the redox status of protein thiols at the cell surface^43, 45^. This evidence supports the possibility that serum γ-GT is not only a marker of inflammation and oxidative stress but also a potential predictor for future HTN^42–45^.

However, the results of previous studies have been inconsistent with regards to the relationship between γ-GT and HTN. Kim *et al*. found a meaningful relationship between high γ-GT levels and HTN only in drinkers^46^, but Stranges *et al*. reported that a higher γ-GT level increased the risk of HTN in both subjects who did and did not drink alcohol^43, 47^. Interestingly, our results showed that serum γ-GT levels did not have a predictive power for future HTN, suggesting a possible different pathophysiology in incident HTN in adolescents. The discrepancies between previous studies on adults and our study may be because our subjects were younger, and because they had low CoVs of γ-GT and fewer deleterious lifestyle factors (such as heavy alcohol consumption, cigarette smoking, and physical inactivity)^46, 47^. Further studies including participants with a wide range of age, different genetic background, insulin resistance status, and inflammatory and oxidative condition are needed to elucidate the true role of γ-GT in predicting HTN.

Even within a normal range of concentration, serum γ-GT has been reported to be related to the presence of diabetes^17, 42, 48^. However, our results did not support serum γ-GT activity as a predictor of T2DM in adolescents. Several possible explanations are as follows: First, epidemiological study on prevalence of diabetes in Taiwan reported that adolescents have less than a 1% prevalence of T2DM^49^. Second, the natural time-course of diabetes is a critical confounding factor while assessing the relationship between metabolic predictors and the development of T2DM. Our subjects were relatively young so that normal glucose levels might be observed at a much earlier age in consideration of ‘compensated period’, i.e., higher secretion of plasma insulin to maintain glucose homeostasis^22^. In support of this, Kong *et al*. have shown high γ-GT levels did not pose a significant risk to dysglycaemia because of their young participants^31^. Finally, our participants were around the age of puberty, and higher levels of sex hormones may have inhibited lipogenesis and improved insulin sensitivity^50^. However, plasma insulin levels parallel to fasting glucose levels were unavailable in this study. Thus we could not evaluate the association between γ-GT and insulin resistance.

The strengths of this study include its longitudinal population-based design and the large number of participants. In addition, this is the first clinical study to identify the optimal CoVs of γ-GT in predicting future MetS in adolescents. Using this simple and widely available biomarker may be helpful in initiating preventive strategies for adolescent MetS. However, there are also several limitations to this study. First, selection bias might exist due to study participants selected from a health screening center rather than from the community. However, the aim of this study was to observe relationships between factors, and thus there should be minimal effects. Second, all subjects of our study were ethnically Chinese, limiting the generalizability of the results to other ethnicities. Finally, data on the levels of serum alanine aminotransferase, insulin, fibrinogen, C-reactive protein, adiponectin and F2-isoprostanes were lacking. Further studies including these parameters and assessing the relationships between γ-GT, systemic oxidative stress, and inflammatory status are needed.

In conclusion, the treatment and prevention of MetS in adolescents has become a public health priority. Our findings suggest that serum γ-GT levels could serve as a clinical predictor for future MetS in adolescents. Using such a low-cost and widely used metabolic biomarker may help pediatricians to screen adolescents at high risk of MetS at an early stage and prevent subsequent deleterious consequences.
