# Author Correction: Predictive Value of Serum Gamma-glutamyltranspeptidase for Future Cardiometabolic Dysregulation in Adolescents- a 10-year longitudinal study

**DOI:** 10.1038/s41598-018-34497-2

**Published:** 2018-11-13

**Authors:** Chien-Ming Lin, Chang-Hsun Hsieh, Chien-Hsing Lee, Dee Pei, Jiunn-Diann Lin, Chung-Ze Wu, Yao-Jen Liang, Yi-Jen Hung, Yen-Lin Chen

**Affiliations:** 1 Department of Pediatrics, Tri-Service General Hospital, National Defense Medical Center, Taipei, Taiwan; 2 Graduate Institute of Medical Sciences, National Defense Medical Center, Taipei, Taiwan; 3 Division of Endocrinology and Metabolism, Department of Internal Medicine, Tri-Service General Hospital, National Defense Medical Center, Taipei, Taiwan; 4 Department of Internal Medicine, Cardinal Tien Hospital, School of Medicine, Fu-Jen Catholic University, New Taipei City, Taiwan; 5 Division of Endocrinology, Department of Internal Medicine, Shuang-Ho Hospital, Taipei Medical University, New Taipei City, Taiwan Republic of China; 6 Division of Endocrinology and Metabolism, Department of Internal Medicine, School of Medicine, College of Medicine, Taipei Medical University, Taipei, Taiwan Republic of China; 7 Department of Life Science, Graduate Institute of Applied Science and Engineering, College of Science and Engineering, Fu-Jen Catholic University, New Taipei City, Taiwan; 8 Department of Pathology, Cardinal Tien Hospital, School of Medicine, Fu-Jen Catholic University, New Taipei City, Taiwan

Correction to: *Scientific Reports* 10.1038/s41598-017-09719-8, published online 29 August 2017

In the original version of this Article, Jiunn-Diann Lin and Chung-Ze Wu were incorrectly affiliated with ‘Division of Endocrinology and Metabolism, Department of Medicine, Shuang-Ho Hospital, Taipei Medical University, New Taipei City, Taiwan’. The correct affiliations are listed below.

“Division of Endocrinology, Department of Internal Medicine, Shuang-Ho Hospital, Taipei Medical University, New Taipei City, Taiwan, ROC”

“Division of Endocrinology and Metabolism, Department of Internal Medicine, School of Medicine, College of Medicine, Taipei Medical University, Taipei, Taiwan, ROC”

In addition, the Acknowledgements section in this Article was incomplete.

“This study was supported by grants from the Tri-Service General Hospital of the National Defense Medical Center (TSGH-C104-024 and TSGH-C105-023), Taiwan.”

now reads:

“This study was supported by grants from the Tri-Service General Hospital of the National Defense Medical Center (TSGH-C104-024, TSGH-C105-023 and TSGHC105-005-S03), Taiwan”

